# Detection of G119S *ace*-*1*^*R*^ mutation in field-collected *Anopheles gambiae* mosquitoes using allele-specific loop-mediated isothermal amplification (AS-LAMP) method

**DOI:** 10.1186/s12936-015-0968-9

**Published:** 2015-12-01

**Authors:** Athanase Badolo, Hironori Bando, Alphonse Traoré, Mami Ko-ketsu, Wamdaogo Moussa Guelbeogo, Hirotaka Kanuka, Hilary Ranson, N’Falé Sagnon, Shinya Fukumoto

**Affiliations:** National Research Centre for Protozoan Diseases, Obihiro University of Agriculture and Veterinary Medicine, Inada-cho, Obihiro, Hokkaido 080-8555 Japan; Centre National de Recherche et de Formation sur le Paludisme (CNRFP), BP 2208, Ouagadougou 01, Burkina Faso; Laboratoire d’Entomologie Fondamentale et Appliquée, Université de Ouagadougou, BP 7021, Ouagadougou 03, Burkina Faso; Department of Tropical Medicine, The Jikei University School of Medicine, Tokyo, 105-8461 Japan; Vector Group, Liverpool School of Tropical Medicine, Pembroke Place, Liverpool, L3 5QA UK

**Keywords:** Insecticide, Resistance, *Ace1*-*R*, LAMP, Malaria, Mosquito

## Abstract

**Background:**

Malaria vectors have developed resistance to the four families of insecticides available for public health purposes. For example, the *kdr* mutation is associated with organochlorines and pyrethroids resistance. It is of particular concern that organophosphate and carbamate resistance associated with the G119S *ace*-*1*^*R*^ mutation has recently increased in West Africa in extent and frequency, and is now spreading through the *Anopheles gambiae* malaria vector population. There is an urgent need to improve resistance management using existing insecticides and new tools to quickly assess resistance level for rapid decision-making.

**Methods:**

DNA extracted from field-collected mosquitoes was used to develop the method. Specific primers were designed manually to match the mutation region and an additional mismatched nucleotide in the penultimate position to increase specificity. Other primers used are common to both wild and mutant types. The allele specific (AS)-LAMP method was compared to the PCR restriction fragment length polymorphism (PCR-RFLP) and real-time PCR (RT-PCR) methods using the genomic DNA of 104 field-collected mosquitoes.

**Results:**

The primers designed for LAMP were able to distinguish between the wild type (*ace*-*1*^*S*^) and mutated type allele (*ace*-*1*^*R*^). Detection time was 50 min for the wild type homozygous and 64 min for the heterozygous. No amplification of the resistant allele took place within the 75-min test period when using the wild type primers. For the *ace*-*1*^*R*^ resistant type, detection time was 51 min for the resistant homozygous and 55 min for the heterozygous. No amplification of the wild type allele took place within the 75-min test period when using the resistant type primers. Gel electrophoresis of LAMP products confirmed that amplification was primer-DNA specific, i.e., primers could only amplify their target specific DNA. AS-LAMP, PCR-RFLP, and RT-PCR showed no significant difference in the sensitivity and specificity of their *ace*-*1*^*R*^ detection ability.

**Conclusions:**

The AS-LAMP method could detect the *ace*-*1*^*R*^ mutation within 60 min, which is faster than conventional PCR-RFLP. This method may be used to quickly detect the *ace*-*1*^*R*^ mutation for rapid decision-making, even in less well-equipped laboratories.

## Background

Recent statistics have revealed worldwide reductions in malaria-related mortality and morbidity, attributable to vector control measures [1]. Although a fair amount of progress has been made on malaria vaccine trials [2], disease prevention remains heavily dependent on insecticide-treated bed nets and indoor residual spraying with insecticides [3].

The resistance of malaria vectors to insecticide is the main concern in vector control interventions. In recent years, resistance to the four classes of insecticides available for public health use has increased across Burkina Faso [4–6]. Resistance to pyrethroid, organophosphates and carbamates has spread in tandem with the increased incidence of the *L1014F kdr* and G119S *ace*-*1*^*R*^ mutations [4, 7]. Recent studies also suggest the importance of metabolic mechanisms in high-level pyrethroid resistance in Burkina Faso [8, 9]. In the case of the *ace*-*1*^*R*^ gene, a single substitution of the amino acid glycine with serine in position 119 (G119S) of this acetylcholinesterase gene is linked to resistance to carbamates and organophosphates insecticides [10]. Furthermore, it has also been reported that the duplication of *ace*-*1* gene in *Anopheles gambiae* (and also *Culex*) contributes to insecticide resistance [11–13].

The latter insecticide families are the only ones still effective against malaria vectors in Burkina Faso [5]. However, molecular investigations have recently confirmed an increase in *ace*-*1*^*R*^ mutations in the population of *An. gambiae*, with an unequal distribution from the western part of the country to the central part, where the mutation still has a very low frequency [4, 14].

The new challenges for malaria control are related to the monitoring of resistance to insecticides, with the goal of better allocating resources for malaria vector control and management of resistance [15]. According to the malERA Consultative Group [16], a related and critical focus of the vector control agenda will be the development of rapid and affordable methods for detecting the emergence of epidemiologically important levels of insecticide resistance. Very relevant in this regard is adapting or developing early resistance detection tools, among which the LAMP (loop-mediated isothermal amplification) method is recognized as a more sensitive and specific method than conventional PCR approaches [17–22].

LAMP has recently been employed to identify the two species of the *An. gambiae* complex [23]. Badolo et al. [24] have developed a single nucleotide polymorphism (SNP)-LAMP method to target the *L1014F* mutation in field-collected mosquitoes. Based on these previous studies, a LAMP-based detection method for the *ace*-*1*^*R*^ mutation in field-collected *An. gambiae* complex is developed herein.

## Methods

### Primer design for LAMP detection of *G119Sace*-*1*^*R*^ mutation in mosquitoes

**Fig. 1:**
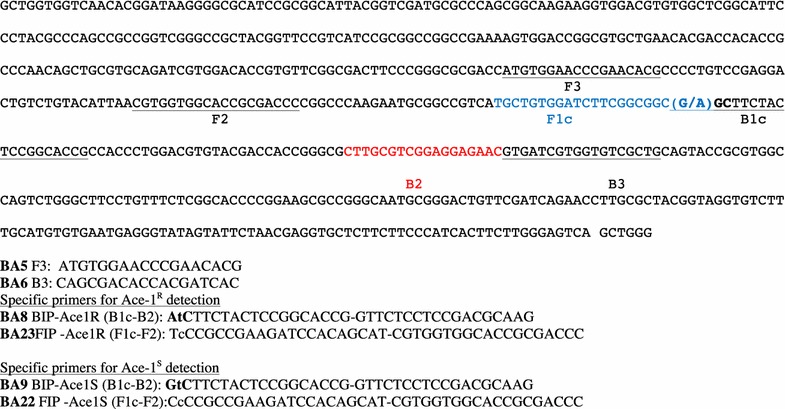
DNA sequence of the acetylcholinesterase gene surrounding the *ace*-*1* ^*R*^ mutation and position of the LAMP primers designed for this study. The mutation nucleotide is *bolded*, G for the wild type (*ace*-*1* ^*S*^) and A for the resistant type (*ace*-*1* ^*R*^). *Lower-case letters* in BA8 and BA9 show additional mismatch

Allele-specific LAMP (AS-LAMP) primers were designed based on published sequences of the acetylcholinesterase gene carrying the *ace*-*1*^*R*^ mutation [10]. To detect the mutant and wild type of *ace*-*1*^*R*^, the specific forward inner primer (FIP) and backward inner primer (BIP) were constructed with the mutation on the 5′ end. An additional mismatched nucleotide was added at the penultimate position of each specific BIP to increase the specificity of the primers to each targeted nucleotide site (Fig. 1). For primer mismatch optimization, DNA fragments from *An. gambiae* mosquitoes carrying either sensitive or resistance-type *ace*-*1* genes were used as the template DNA of the LAMP reaction. The outer primers (B3 and F3) were identical for the two primer sets.

### Mosquito collection

All mosquitoes used in this study were collected during the rainy season of 2008 in the village of Soumousso (N 11°00, W 04°03) located in the Sudan Savannah zone, 380 km from Ouagadougou and 38 km from Bobo-Dioulasso. The village is located within the cotton belt, where insecticide pressure is higher than in other parts of the country, as is the frequency of *ace*-*1*^*R*^ [4]. Mosquitoes were collected as larvae and reared to adults for morphological classification of species and sex. Only the female *An. gambiae* complex mosquitoes were used for analysis. The mosquitoes were kept in silica gel containing tubes until used.

### Extraction of DNA from field-collected mosquitoes

The genomic DNA of field-collected mosquitoes was extracted by homogenizing individual mosquitoes with a pellet pestle in 100 µl Buffer A [0.1 M Tris HCl (pH 9.0), 0.1 M EDTA, 1 % SDS, and 0.5 % diethyl pyrocarbonate] and incubating the homogenate for 30 min at 70 °C. After incubation, 22.4 µl of 5 M potassium acetate was added, and the mixture was cooled on ice for 30 min. After centrifugation at 15,000 rpm for 15 min at 4 °C, the DNA-containing supernatant was transferred into a new tube and mixed with 45 µl isopropanol. The solution was centrifuged at 15,000 rpm for 20 min at 4 °C, and the supernatant was discarded. The DNA pellet was rinsed with 70 % ethanol and dried. The pellet was then diluted in 30 µl TE buffer.

### LAMP reaction procedure

The LAMP reaction was carried out following manufacturer recommendations (Eiken Chemicals, Tokyo, Japan). A master mix was prepared using 6.25 µl of 2× reaction mix (2.8 mM each of dNTP, 40 mM Tris–HCl (pH 8.8), 20 mM KCl, 16 mM MgSO_4_, 20 mM (NH_4_)_2_SO_4_, 0.2 % Tween 20, and 1.6 M betain), 2.75 µl of distilled water and 0.5 µl of each of the primers and the *Bst* DNA polymerase. The concentration of the primers used was 40 pmol/µl for the inner primers (BIP and FIP) and 5 pmol/µl for the outer primers (B3 and F3). A volume of 11.5 µl of the master mix was placed into test tubes (including a negative control). One µl of DNA solution was added to the sample tubes and 1 µl of distilled water to the negative control tube. All procedures were carried out on ice. The tubes were then incubated in a water bath or LA-200 real-time turbidimeter (Eiken Chemicals) at 63 °C, and turbidity was measured. The reaction was terminated by heating the tube at 95 °C for 5 min. A 2 % agarose gel electrophoresis at 100 V of the LAMP products was performed and the gel was stained with ethidium bromide for examination under UV light to check amplification bands.

### PCR-RFLP for *ace*-*1*^*R*^ mutation detection

The MR4 protocol [25] (based on Weil et al. [10]) was used for *ace*-*1*^*R*^ mutation detection method by PCR-RFLP analysis. The *ace*-*1*^*R*^ SNP region was amplified with two primers (MOUSTDIR1:) (25 pmol/μl) [CCGGGNGCSACYATGTGGAA] MOUSTREV1 (25 pmol/μl) [ACGATMACGTTCTCYTCCGA]. PCR was carried out in a 25-µl tube using KOD FX Neo DNA polymerase (Toyobo, Osaka, Japan). Amplification was performed with 1 µl of genomic DNA as the template using the following PCR programme: 94 °C/2 min × 1 cycle, (98 °C/10 s, 68 °C/30 s) × 35 cycles, 68 °C/2 min × 1 cycle, and 4 °C hold. The PCR product was digested by adding 1 μl AluI (New England Biolabs, Ipswich, MA, USA) restriction enzyme, 2 μl of H_2_0, and 2 μl of buffer to 15 μl. An incubation at 37 °C for 8–24 h [25] was observed. Finally, 5 µl of the digestion product was run on 2 % agarose gel and stained in ethidium bromide.

### Real-time PCR (RT-PCR) detection of the *ace*-*1*^*R*^ mutation

RT-PCR detection of *ace*-*1*^*R*^ was carried out based on the methods in the *Anopheles* Research protocol [25, 26], with the primers (forward: GCCGTCATGCTGTGGATCTT, reverse: GCCCGGTGGTCGTACAC) and probes (wild type allele: VIC-CGGCGGCTTCTAC, mutant type allele: FAM-CGGCAGCTTCTAC) with TaqMan GTXpress master mix (Life Technologies, Carlsbad, CA, USA) and analysed by StepOne RT-PCR machine (Life Technologies). For the positive controls, DNA fragments from *An. gambiae* mosquitoes carrying either sensitive or resistance-type *ace*-*1* genes cloned into pBSSK vectors were used. PCR alignment scores were taken from respective endpoint scatter plots.

### Sequencing of mosquito DNA

DNA from *An. gambiae* mosquitoes was used as PCR templates. The target sequence was amplified using the PCR primers BA3 (GCTGGTGGTCAACACGGATA) and BA4 (CCCAGCTGACTCCCAAGAAG), designed to amplify a 603-bp DNA fragment of *ace*-*1* including its SNP region. PCR was performed in a tube with KOD FX Neo DNA polymerase (0.5 µl). The PCR programme was 94 °C/2 min × 1 cycle, (98 °C/10 s, 68 °C/30 s) × 35 cycles, 68 °C/2 min × 1 cycle, and 4 °C hold. The PCR product was purified using a geneclean kit (MP Biomedicals, Santa Ana, CA, USA) and the final product was diluted in 10 µl of distilled water. The nucleotide sequences were determined with a BigDye terminator sequencing kit v. 3.1 (Life Technologies, Carlsbad, CA, USA) using an automated 3100 genetic analyzer (Life Technologies).

### Statistical analysis

Sensitivity was defined as the ratio of true positives to combined true and false positives, and specificity as the ratio of true negatives to combined true negatives and false negatives. Calculation of specificity and sensitivity confidence limits of AS-LAMP and RT-PCR was carried out using the Wilson score method [27].

## Results

### Primer sequences

The BIP and FIP primers, containing the SNP with an additional mismatched nucleotide attached to the penultimate nucleotide at the 3′ (BIP) or 5′ end (FIP), were designed as specific primers to detect the G119S single nucleotide by AS-LAMP. The two specific primers and the mismatched nucleotide increase the specificity of both primers for their target DNA. The different regions targeted by the primers and the primer sequences are shown in Fig. 1.

### Detection of the *ace*-*1*^*R*^ mutation by AS-LAMP using genomic mosquito DNA

**Fig. 2:**
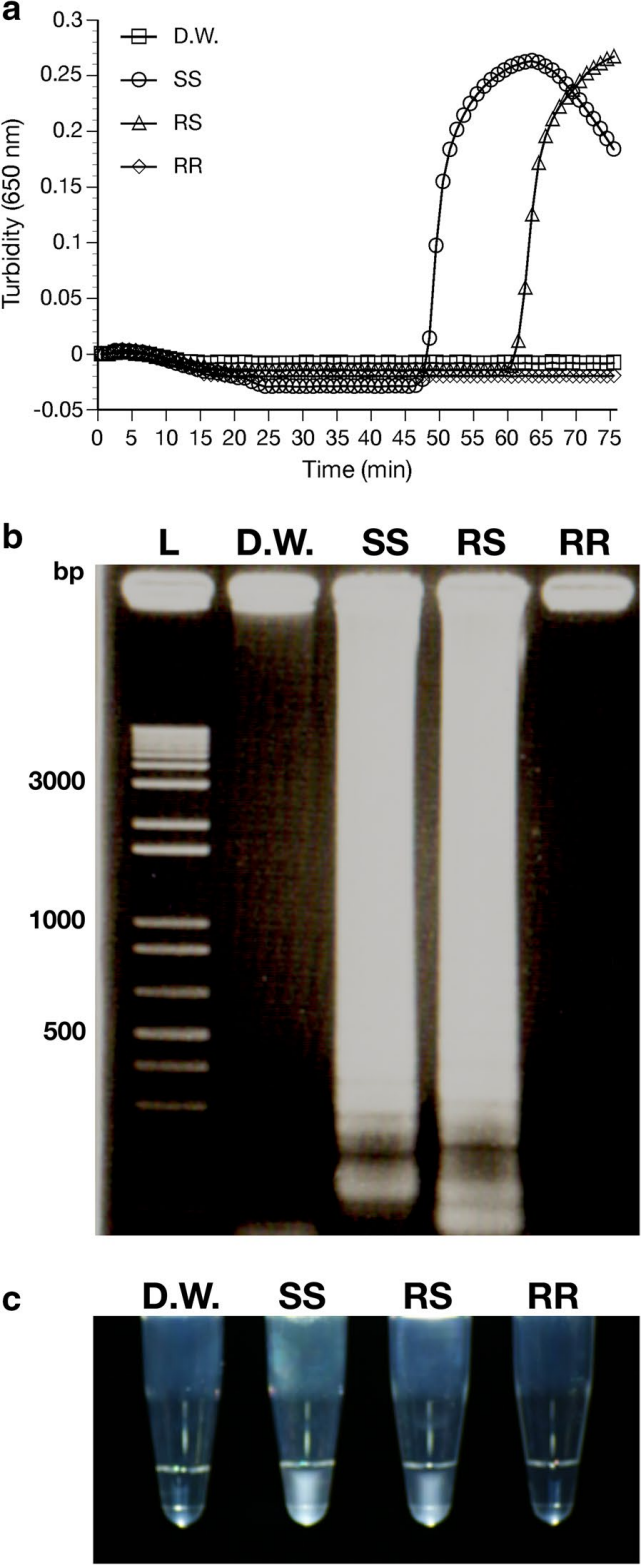
Wild type (*ace*-*1* ^*S*^) mutation detection using *ace*-*1* ^*S*^ specific primers and mosquito genomic DNA. Amplification graph (**a**), gel electrophoresis (**b**) and naked eye detection (**c**) using wild type primers against negative control (*D.W.* distilled water), *SS* wild type homozygous DNA, *RS* heterozygous, *RR* *ace*-*1* ^*R*^ type homozygous, *L* DNA ladder

The primers designed for AS-LAMP were able to distinguish between the wild type allele (*ace*-*1*^*S*^) and the mutated type allele (*ace*-*1*^*R*^). Detection time for the wild type mutation was ~ 50 min after incubation in the turbidimeter using the wild type primers; detection time for heterozygous samples was 64 min using the same primers. Using these primers, no amplification of the resistant allele (*ace*-*1*^*R*^) took place within the 75-min test period (Fig. 2a).

**Fig. 3:**
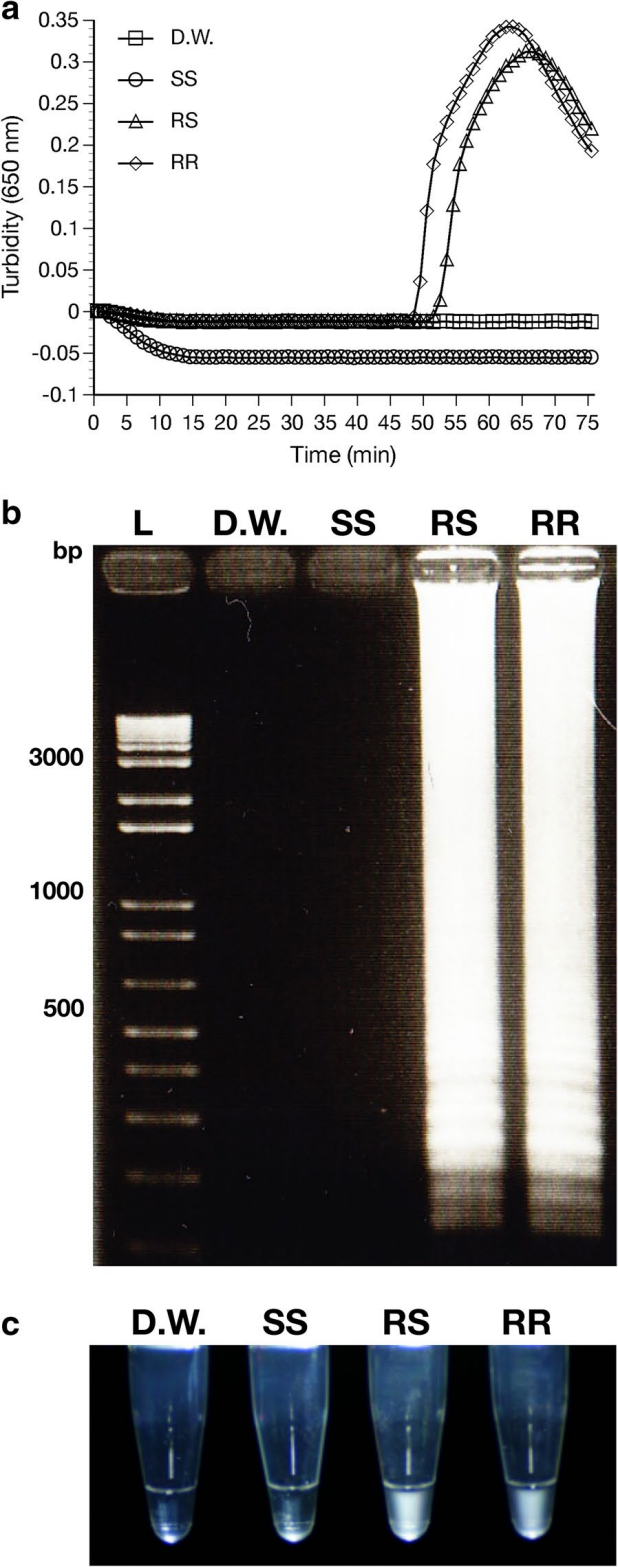
Mutant type (*ace*-*1* ^*R*^) mutation detection using *ace*-*1* ^*R*^ specific primers and mosquito genomic DNA. Amplification graph (**a**), gel electrophoresis (**b**) and naked eye detection (**c**) using *ace*-*1* ^*R*^ type primers against negative control (*D.W.* distilled water), *SS* wild type homozygous DNA, *RS* heterozygous, *RR* *ace*-*1* ^*R*^ type homozygous, *L* DNA ladder

For *ace*-*1*^*R*^ resistant type mutation detection, amplification started at ~51 min after incubation for resistant homozygous samples and at ~55 min for heterozygous samples, with no amplification of the wild type (*ace*-*1*^*S*^) taking place within the 75-min test period when using the resistant type primers (Fig. 3a). Gel electrophoresis of the LAMP products confirmed that the amplification was primer DNA-specific, i.e., the primers could only amplify their target-specific DNA (Figs. 2b, 3b).

### PCR-RFLP and real-time RT-PCR identification of the *ace*-*1*^*R*^ mutation

PCR-RFLP detection based on the protocol of Weill et al. [10] was used to detect the *ace*-*1*^*R*^ mutation in 104 field-collected mosquitoes. Three homozygous resistant samples, nine heterozygous samples and 95 sensitive homozygous samples were identified.

**Fig. 4:**
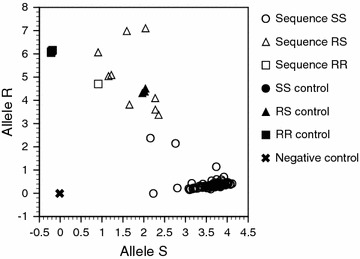
RT-PCR identification of the *ace*-*1* ^*R*^ mutation. Scatter plots of the fluorescence of *ace*-*1* ^*R*^ mutation detection. *Sequence SS* the DNA samples identified as sensitive homozygous by the sequencing method, *sequence RS* identified as heterozygous, *sequence RR* identified as resistant homozygous, *SS control* the plasmid coding the *ace*-*1* ^*S*^ sequence, *RS control* mixture of the plasmid coding the *ace*-*1* ^*S*^ sequence and that coding the *ace*-*1* ^*R*^ sequence, *RR control* the plasmid coding the *ace*-*1* ^*R*^ sequence, *negative control* distilled water instead of template DNA

RT-PCR detection of *ace*-*1*^*R*^, based on the methods in the *Anopheles* Research protocol [25], found 12 heterozygous samples and 92 homozygous samples for the sensitive alleles, while failing to clearly distinguish between any of the homozygous versus heterozygous samples (Fig. 4; Table 1).

**Table 1:** Comparing the detection of the *ace*-*1* ^*R*^ mutation using AS-LAMP and PCR-RFLP

Genotype	Number tested					Sensitivity (95 % CL)			Specificity (95 % CL)	
	Sequencing	PCR-RFLP	RT-PCR	AS-LAMP	PCR-RFLP	RT-PCR	AS-LAMP	PCR-RFLP	RT-PCR	AS-LAMP
Ace1RR	1	1	0	1	1	0	1	1	1	1
					(0.21–1.00)	(0–0.79)	(0.21–1.00)	(0.96–1)	(0.96–1)	(0.96–1)
Ace1RS	9	9	12	8	1	0.75	0.89	1	1	1
					(0.79–1.00)	(0.47–0.91)	(0.56–0.98)	(0.96–1)	(0.96–1)	(0.96–1)
Ace1SS	94	94	92	95	0.99	0.98	1	0.90	1	0.90
					(0.94–0.99)	(0.93–0.99	(0.96–1)	(0.60–0.98)	(0.72–1)	(0.60–0.98)
Total	104	104	104	104	–		–	–		–

Genotypes and associated number of mosquitoes detected using PCR-RFLP, RT-PCR, and AS-LAMP methods, compared to sequencing results. Percent sensitivity and specificity of each method relative to the sequencing are in parentheses

### Sensitivity and specificity of PCR-RFLP, RT-PCR and AS-LAMP methods relative to sequencing

To investigate the comparative sensitivity for detecting each genotype of the *ace*-*1*^*R*^ mutation, *ace*-*1*^*R*^ mutation detection using the AS-LAMP, PCR-RFLP and RT-PCR methods for 104 mosquitoes was performed. The samples were sequenced first using standard PCR (Table 1): these sequences were treated as the ‘gold standard’ for identification. The same samples were then analysed using AS-LAMP, PCR-RFLP and RT-PCR, and the respective results were compared against the corresponding standard PCR-derived sequence. AS-LAMP and PCR-RFLP had the same sensitivity for the homozygous resistant type [100 %, confidence limit (CL) 21–100 %], while RT-PCR could not distinguish clearly any resistant specimen (Fig. 4). For detection sensitivity of the heterozygous type, there was no statistically significant difference between AS-LAMP (89 %, CL 56–99 %), PCR-RFLP (100 %, CL 79–100 %), and RT-PCR (75 %, CL 47–91 %), although the sample number may have been too few to detect significance. The specificity of all three methods was perfect (100 %) for resistant homozygous and heterozygous detection. There were, however, slight (albeit not statistically significant) differences in detection specificity for the sensitive homozygous type (PCR-RFLP and AS-LAMP 90 %, CL 60–98 %; RT-PCR 100 %, CL 72–100 %).

### AS-LAMP method detection using water bath and naked eye detection

To evaluate whether the AS-LAMP reaction could be done with only a water bath and scored by eye, without a real-time turbidimeter, 24 DNA samples used in Table 1 were randomly chosen and subjected to AS-LAMP with a water bath. As shown in Table 2, the naked eye detection results of all samples were completely identical to that obtained with the real-time turbidimeter.

**Table 2:** Comparison of the result of AS-LAMP method using water bath and naked eye detection with real-time turbidimeter

Real-time turbidimeter	Water bath		
	Ace1RR	Ace1RS	Ace1SS
Ace1RR	0	0	0
Ace1RS	0	2	0
Ace1SS	0	0	22

## Discussion

Resistance to the four classes of insecticide available for public health use (organochlorines, organophosphates, carbamates, and pyrethroids) has been found in *Anopheles* species in different parts of Africa [28–30]. This resistance may negatively impact the efficacy of insecticide-treated bed nets (ITNs) and indoor residual spraying (IRS) [31]. As no other classes of insecticides are available at this time, managing current insecticides seems to be the key solution to vector control.

Developing easy-to-use tools to detect epidemiologically significant resistant levels is a prerequisite for vector control [15]. A more sensitive and specific method that can be used with minimum equipment can facilitate decision-making on insecticide use.

In the present study, an AS-LAMP method targeting the *ace*-*1*^*R*^ mutation, which is involved in resistance to carbamates and organophosphates in *An. gambiae* malaria vectors, was developed. Primers designed with a mutation on the 3′ end of the BIP primer and an additional mismatched nucleotide (following Badolo et al. [24]) were unable to distinguish between the *ace*-*1*^*S*^ and *ace*-*1*^*R*^ mutation. The sequence of the acetylcholinesterase gene on which used was rich in GC and impeded the designing of suitable primers. Specificity to distinguish between *ace*-*1*^*S*^ and *ace*-*1*^*R*^ increased when the mutations were appended to the two inner primers.

Primers were able to detect heterozygous samples in less than 64 min, and the detection method proved to be as sensitive as and of comparable specificity to PCR-RFLP, although the tests of sensitivity were underpowered because the number of individuals with resistant alleles was very low. Identification of the *ace*-*1*^*R*^ mutation using the AS-LAMP method has several advantages. Since the LAMP method can be performed with only a water bath and the result can be interpreted by the naked eye, gel electrophoresis analysis is not essential. This method could allow the national malaria control programmes in less developed countries to monitor *ace*-*1*^*R*^ mutation incidence on a local level and to facilitate insecticide selection for bed net treatment or indoor use. In this study, AS-LAMP operated with a water bath actually obtained the same result as with a turbidimeter, although the number of samples tested was limited. A turbidimeter is extremely useful when establishing novel LAMP methods because it provides real-time information of amplification, although some set-up cost is required (approximately two times or less that of a standard thermal cycler). However, this machine is not essential in routine examinations using LAMP.

Another advantage of LAMP is its high reaction speed: it is actually similarly fast to RT-PCR and faster than PCR-RFLP. The times required of AS-LAMP and PCR prior to RFLP analysis are similar as well. However, RFLP analysis requires another few hours for restriction digestion and gel electrophoresis analysis. Furthermore, the restriction digestion time used in this study was chosen to follow the MR4 protocol [25], but on the longer side of the manufacturer’s suggestion (1 h) in order to prevent incomplete digestion.

A problem with the PCR-RFLP-based method, and presumably the AS-LAMP method also, is its inability to even tentatively detect duplication of the *ace*-*1* gene, which is increasingly common and linked to resistance to insecticide [11–13]. The RT-PCR method does not do this perfectly, but can nonetheless provide quantitatively useful information [26]. In this study, RT-PCR could not distinguish clearly the resistant homozygous versus the heterozygous genotypes. This might have been caused by gene duplication, although this is only speculation and was not confirmed here.

Resistance to insecticides requires regular monitoring. The tested method can readily be used for detecting the *ace*-*1*^*R*^ mutation with minimum equipment, and may serve as an alternative to PCR-RFLP for resistance monitoring in less equipped laboratories and for quick decision-making. Further development of LAMP-based detection kits or chips could be revolutionary tools for malaria vector control in developing countries by detecting epidemiologically important resistance to insecticide level at the site of transmission.

## Conclusion

The AS-LAMP method with both FIP and BIP specific primers to detect the *ace*-*1*^*R*^ mutation in *An. gambiae* is similar to the PCR method in terms of specificity and sensitivity, with the added advantage of less required facilities.
